# Supplementary material for: Meta-Analysis and Gene Set Enrichment Relative to ER Status Reveal Elevated Activity of MYC and E2F in the “Basal” Breast Cancer Subgroup
Source: PLoS One. 2009 Mar 9;4(3):e4710. doi: 10.1371/journal.pone.0004710 (PMC2650420; doi:10.1371/journal.pone.0004710)
Supplement: Dataset S1 — GSEA results from first screen. The file contains a zipped archive of the HTML results of the second GSEA screen. Reports for enriched or depleted datasets may be accessed from the “index.html” link within the my_analysis.GseaPreranked.1219975950625.rpt.zip ZIP archive. Enrichment plots are available for each of the 50 most enriched and depleted gene sets. (13.32 MB ZIP) [file pone.0004710.s001.zip › my_analysis.GseaPreranked.1219975950625.rpt.zip!//FRASOR_ER_UP.html]

Details for gene set FRASOR\_ER\_UP[GSEA]

|  || Dataset | zSco.ERneg.to.ERpos\_collapsed |
| Phenotype | NoPhenotypeAvailable |
| Upregulated in class | na\_neg |
| GeneSet | FRASOR\_ER\_UP |
| Enrichment Score (ES) | -0.63313746 |
| Normalized Enrichment Score (NES) | -2.277505 |
| Nominal p-value | 0.0 |
| FDR q-value | 8.847091E-5 |
| FWER p-Value | 0.0010 |
Table: GSEA Results Summary

  

Fig 1: Enrichment plot: FRASOR\_ER\_UP      
 Profile of the Running ES Score & Positions of GeneSet Members on the Rank Ordered List

  

| PROBE | GENE SYMBOL | GENE\_TITLE | RANK IN GENE LIST | RANK METRIC SCORE | RUNNING ES | CORE ENRICHMENT || 1 | RFC4 |  |  | 370 | 4.741 | 0.0291 | No |
| 2 | MYBL1 |  |  | 1787 | 2.704 | -0.0450 | No |
| 3 | CDC6 |  |  | 3185 | 1.847 | -0.1279 | No |
| 4 | PEG10 |  |  | 3478 | 1.718 | -0.1292 | No |
| 5 | TOP2A |  |  | 3735 | 1.610 | -0.1291 | No |
| 6 | RASGRP1 |  |  | 4559 | 1.300 | -0.1754 | No |
| 7 | SDC2 |  |  | 5140 | 1.101 | -0.2059 | No |
| 8 | GREB1 |  |  | 6264 | 0.727 | -0.2816 | No |
| 9 | PTGES |  |  | 7474 | 0.319 | -0.3688 | No |
| 10 | CALCR |  |  | 7590 | 0.273 | -0.3741 | No |
| 11 | AREG |  |  | 8345 | -0.014 | -0.4307 | No |
| 12 | AP1G1 |  |  | 9050 | -0.336 | -0.4796 | No |
| 13 | ADCY9 |  |  | 10125 | -0.918 | -0.5494 | No |
| 14 | EPB41L3 |  |  | 10414 | -1.097 | -0.5579 | No |
| 15 | CXCL12 |  |  | 11393 | -1.923 | -0.6084 | Yes |
| 16 | WISP2 |  |  | 11475 | -2.007 | -0.5904 | Yes |
| 17 | GLRB |  |  | 11635 | -2.152 | -0.5765 | Yes |
| 18 | FOS |  |  | 12389 | -3.085 | -0.5961 | Yes |
| 19 | RAB31 |  |  | 12488 | -3.244 | -0.5645 | Yes |
| 20 | NRIP1 |  |  | 12554 | -3.341 | -0.5292 | Yes |
| 21 | MAPT |  |  | 12626 | -3.480 | -0.4928 | Yes |
| 22 | RET |  |  | 12638 | -3.506 | -0.4515 | Yes |
| 23 | CA12 |  |  | 12705 | -3.627 | -0.4129 | Yes |
| 24 | TFF1 |  |  | 12775 | -3.809 | -0.3723 | Yes |
| 25 | SIAH2 |  |  | 13112 | -4.869 | -0.3391 | Yes |
| 26 | SLC39A6 |  |  | 13151 | -5.039 | -0.2815 | Yes |
| 27 | IGFBP4 |  |  | 13227 | -5.654 | -0.2192 | Yes |
| 28 | PDZK1 |  |  | 13242 | -5.783 | -0.1508 | Yes |
| 29 | EGR3 |  |  | 13250 | -5.968 | -0.0796 | Yes |
| 30 | SLC22A5 |  |  | 13310 | -7.063 | 0.0008 | Yes |
Table: GSEA details [plain text format]

  

Fig 2: FRASOR\_ER\_UP: Random ES distribution      
 Gene set null distribution of ES for **FRASOR\_ER\_UP**
